# Retraction: Specific clones of *Trichomonas tenax* are associated with periodontitis

**DOI:** 10.1371/journal.pone.0312329

**Published:** 2024-10-14

**Authors:** The PLOS ONE Editors

The *PLOS ONE* Editors retract this article [1, 2] due to concerns about compliance with the PLOS Human Subjects Research policy.

Specifically, the ethics statement of this article [1] states that the study was approved by the clinical research ethics committee of the Institute Fédératif de Recherche (IFR) 48, Aix-Marseille University, under protocol N° 2016–011. The reported ethics approval document was issued in September, 2016, after the reported study period (January 2015 –June 2016). PLOS does not accept retrospective ethics approval.

Furthermore, document N° 2016–011 grants approval for the collection of stool samples only, as opposed to the subgingival dental plaque samples reported in this article [1], and it is unclear whether the approval covers collection of control samples from healthy participants.

In addition, PLOS identified potential competing interests between the approving ethics committee and one or more of the article’s authors, and the ethics approval cited in the article’s Ethics Statement has been reused across numerous articles addressing different research objectives. A summary of articles reporting the ethics approval reference N° 2016–011 that PLOS is aware of is available in the S1 File provided with this notice.

A representative of the Aix-Marseille Université Ethics Committee stated that the institute disagrees with the retraction decision. They confirmed that the reference to the approval document N° 2016–011 in this article [1] is an error, but they stated that this study is a retrospective study that does not involve the recruitment of human subjects as dental calculus was collected during routine care, the samples are considered to be waste and this study is not considered to be research involving the human person that would require approval from a Comité de Protection des Personnes (CPP).

Neither the institute nor the authors provided documentation that confirms prospective ethics approval for the collection of the subgingival dental plaque samples from healthy participants and patients with periodontitis, as reported in this article [1].

JA and BLS did not agree with the retraction. SB, AG, ET, MP, HC, and GA either did not respond directly to the retraction decision or could not be reached.

## Supporting information

S1 File List of 55 articles referencing ethics approval number N° 2016–011. (XLSX)
